# Detection of tandem repeats in the *Capsicum annuum* genome

**DOI:** 10.1093/dnares/dsad007

**Published:** 2023-04-25

**Authors:** Valentina Rudenko, Eugene Korotkov

**Affiliations:** Institute of Bioengineering, Research Center of Biotechnology of the Russian Academy of Sciences, Moscow 119071, Russia; Institute of Bioengineering, Research Center of Biotechnology of the Russian Academy of Sciences, Moscow 119071, Russia

**Keywords:** repeats, genetic algorithm, sequence, *Capsicum annuum*

## Abstract

In this study, we modified the multiple alignment method based on the generation of random position weight matrices (RPWMs) and used it to search for tandem repeats (TRs) in the *Capsicum annuum* genome. The application of the modified (m)RPWM method, which considers the correlation of adjusting nucleotides, resulted in the identification of 908,072 TR regions with repeat lengths from 2 to 200 bp in the *C. annuum* genome, where they occupied ~29%. The most common TRs were 2 and 3 bp long followed by those of 21, 4, and 15 bp. We performed clustering analysis of TRs with repeat lengths of 2 and 21 bp and created position-weight matrices (PWMs) for each group; these templates could be used to search for TRs of a given length in any nucleotide sequence. All detected TRs can be accessed through publicly available database (http://victoria.biengi.ac.ru/capsicum_tr/). Comparison of mRPWM with other TR search methods such as Tandem Repeat Finder, T-REKS, and XSTREAM indicated that mRPWM could detect significantly more TRs at similar false discovery rates, indicating its superior performance. The developed mRPWM method can be successfully applied to the identification of highly divergent TRs, which is important for functional analysis of genomes and evolutionary studies.

## 1. Introduction

In eukaryotic organisms, a considerable part of the genome is occupied by dispersed and tandem repeat (TR) sequences. In the human and plant genomes, repeats, and transposable elements constitute about 43% and 80%, respectively.^1^ TRs can be found in the promoters, 3ʹ untranslated regions, exons, and introns, and it is suggested that repeats are involved in DNA compaction and evolutionary events through contribution to gene expression divergence.^2,3^ Most TRs can mutate quickly, thus adding to genetic variability and promoting rapid adaptation of organisms to new environmental conditions.^4,5^ TRs present in centromeric, pericentromeric, and telomeric regions^6–9^ and areas of chromosome breakage can lead to chromosomal rearrangements.

TRs are generally classified into microsatellites (or short TRs [STRs]) and minisatellites with repeat lengths of 1–6 and 7–60 bp, respectively. TRs longer than 60 bp (and even longer than 1,000 bp) are also observed.^10^ Repeat sequences may be species-specific, and their numbers could vary considerably owing to DNA polymerase slippage and unequal recombination.^11^ Short TRs are detected at the ends of transposable elements: SINEs, LINEs, and retrotransposons, where they affect the possibility of transposition, thus exerting genetic regulation. It is noted that more than 25 human hereditary diseases are associated with STRs,^12^ including fragile X syndrome^13,14^ and autism.^15,16^

There are cases of correlation between the TR length and function. Thus, 3 bp period is characteristic for DNA coding regions and is associated with the triplet nature of the codons,^17^ whereas TRs of 10–11 bp (referred to as 10.5 bp) has been demonstrated in nucleosomal DNA.^18,19^ TRs of 5–7 and 11–14 bp are characteristic for enhancers and other non-coding regions adjacent to genes, whereas long (120.9 bp) TRs are observed at the 5ʹ ends of the transcripts.^20^

The diverse regulatory roles of TRs have spurred the development of computer programs for TR search in the genomes.^21^ Some of them can identify TRs with insertions or deletions (indels), provided that the degree of similarity between repeats is high (>50%). For example, Tandem Repeat Finder (TRF)^22^ searches the candidates based on the statistics of k-tuple matches, and the TR is recreated considering possible indels, when k-tuples are placed relative to each other. As the presence of indels within k-tuples complicates TR detection, in the TRStalker algorithm,^23^ gapped q-grams allowing indels are tracked, resulting in the detection of long (>10 bp), more divergent repeats. T-REKS^24^ and XSTREAM^25^ using dynamic programming to build multiple alignments can also find TRs with indels. However, these methods cannot detect TRs with similarity < 40%.

Highly divergent (fuzzy) TRs are of particular interest because of their presence in gene regulatory regions, where they interact with transcription factors.^26^ Fuzzy TRs can be identified with the methods that use information decomposition^21,27^ and the Fourier transform (FT).^20,28,29^ Such approaches have revealed that short-length (2, 3, 4, and 6 bp) repeats are characteristic for introns and exons.^30^ However, the disadvantages of the FT and information decomposition methods are that indels are not considered.

Thus, there is currently a need for a universal method that can identify long TRs with a large number of accumulated indels. In our previous studies, we have developed the random position weight matrix (RPWM) method, which could effectively detect TRs with the average number of mutations per nucleotide (x) up to 3.2.^31,32,33^ Here, we improved the RPWM method by considering the correlation between adjacent DNA bases, which allowed identification of TRs with 2–200 bp, and refined the estimation of the statistical significance of the found TRs by using the Monte Carlo method. As a result, it was possible to increase the average number of TR-containing regions detected per 10^6^ DNA bases by 1.5 times.

The modified (m) RPWM method was used to search for TRs in the pepper (*Capsicum annuum*) genome. Although the genome of *C. annuum* was sequenced in 2014,^34^ no detailed analysis of its TRs has been performed except for the study showing that 15.64% of expressed sequence tags contain STRs (2–6 bp)^35^ which have been subsequently used to develop various polymorphic microsatellite markers.^36^ By applying the mRPWM method, we identified all possible TRs with a length 2–200 bp in the *C. annuum* genome and created a corresponding database (http://victoria.biengi.ac.ru/capsicum_tr/).

## 2. Materials and methods

### 2.1. RPWM algorithm

The pepper (*Capsicum annuum*) genome was used as a model to search for TRs with the improved RPWM algorithm. RPWM, which uses position weight matrices (PWMs),^33^ was modified to consider the correlation of adjacent nucleotides in TR detection and more accurately determine the statistical significance of the identified TRs. In order to understand the nature of the improvement, let us review the main steps of the RPWM algorithm.

Step1. A window with length *L* = 650 b denoted as sequence *S* moved along a chromosome with a step of 10 b, and TRs of length *n* = 2–50 b were searched for in each *S*.

Step 2. A set of 500 random PWMs with dimensions of *n* × 4 (TR length × 4 DNA bases) was generated and denoted as *Q*_*n*_; *N*_*o*_ = 500 is the volume of set *Q*_*n*_. Each matrix *Q*_*n*_(*i*) was transformed so that sum $\sum_{i=1}^{n}\sum_{j=1}^{4}pwm{\left(i,j\right)}^{2}$ was always constant. In this formula, *pwm*(*i*,*j*) is the element of the PWM on row *i* and column *j*, *p*_1_(*i*) = 1/_*n*_, and *p*_2_(*j*) = *N*(*j*)/*L*, where *N*(*j*) is equal to the number of A, T, C, or G bases in sequence *S*. Sum $\sum_{i=1}^{n}\sum_{j=1}^{4}pwm(i,j)p_{1}(i)p_{2}(\;j)$ was also kept constant for all matrices from set *Q*_*n*_. This transformation was necessary to ensure that similarity function *Fmax* (step 3 below) had similar distribution for different *n*^37^; otherwise, it would be very difficult to find the most statistically significant period and determine TR length in sequence *S*.

Step 3. For each set *Q*_*n*_, we applied the genetic algorithm to find matrix $Q_{n}^{\max}$ with the best local alignment to sequence *S*; the maximum value of similarity function *F*_*max*_ was used as the object function and matrices from set *Q*_*n*_ – as organisms. The genetic algorithm was applied as follows. A. Each matrix from set *Q*_*n*_ was locally aligned to sequence *S* as previously described,^33,38^ and *F*_*max*_ and local alignment coordinates in sequence *S* were obtained for each matrix, which allowed accurate determination of TR coordinates in sequence *S*; *F*_*max*_ values were written in vector *V*_*n*_(*i*) (*i* = 1, 2,…, *N*_*q*_). B. Vector *V*_*n*_(*i*) was ranked in a descending order so that *V*_*n*_(1) contained the maximum value of *F*_*max*_, and random mutations were introduced into 10 randomly selected matrices from set *Q*_*n*_ by replacing one randomly selected cell with a random number from −10 to +10. Two matrices were randomly selected as ‘parents’ and randomly superposed to create a new ‘descendant’ matrix; then, a matrix with the smallest *F*_*max*_ value (contained in *V*_*n*_(500)) was removed from set *Q*_*n*_. Then, we went back to point A and recalculated all *V*_*n*_ values for the mutated matrices and the ‘descendant’; for the matrices that did not change in point B, local alignment was not performed and *V*_*n*_ was not recalculated. The A–B cycle was repeated until *V*_*n*_(1) tended to increase. As a result, we obtained matrix *V*_*n*_(1) and local alignment of sequence *S* for a TR of length *n*.

Step 4. Steps 2–3 were performed for *n* = 2–50 bases to calculate all *V*_*n*_(1) values and determine *n* with the maximum *V*_*n*_(1) value, which was designated as *n*_*max*_(*k*), where coordinate *k* is the beginning of the window (sequence *S*) in the chromosome (step 1). Thus, for each window, we obtained *n*_*max*_(*k*), $V_{n_{\max}(k)}\left(1\right)$, matrix $Q_{n}^{\max}$, and local alignment of matrix $Q_{n}^{\max}$ and sequence *S*.

Step 5. Windows in sequence *S* were shifted by 10 bases to overlap with each other, and $V_{n_{\max}(k)}\left(1\right)$ values were filtered as follows: the dependence of *V(1)* on the TR length (*n*) was excluded, resulting in $Vec(k)=V_{n_{\max}(k)}\left(1\right)$, and the local maxima in vector *Vec*(*k*) were chosen so that *Vec*(*k*) > *V*_0_ and *Vec*(*k-i*) ≤ *Vec*(*k*) ≥*Vec*(*k+i*), where *i* ranges from 1 to 60. The selected local maxima represented the final result. Thus, for each local maximum, we obtained *n*_*max*_(*k*), *Vec*(*k*), matrix $Q_{n}^{\max}$, and local alignment of matrix $Q_{n}^{\max}$ and sequence *S*.

Step 6. A random sequence *S* was used to select threshold level *V*_0_ for local maxima *Vec*(*k*). Steps 1–5 were repeated for this sequence, and a set of local maxima *Vec*_*r*_(*k*) was obtained; the numbers of local maxima *Vec*(*k*) and *Vec*_*r*_(*k*) were denoted as *N*_*v*_ and *N*_*r*_, respectively. Then, we chose *V*_0_ with the ratio *N*_*r*_(*Vec*(*k*) > *V*_0_)/ *N*_*v*_(*Vec*(*k*) > *V*_0_) ~ 0.027. These local maxima and the corresponding *n*_*max*_(*k*), matrix $Q_{n}^{\max}$ together with local alignment of the matrix and sequence *S* represented the final result of the algorithm.

### 2.2. Modified RPWM algorithm

#### 2.2.1 Calculation of statistical significance *Z*(*n*)

We modified RPWM^33^ to consider the statistical significance of the identified TRs. Matrices *Q*_*n*_(*i*) transformed in section 2.1 allowed comparison of *V*_*n*_(1) for different *n*, which could be performed if *F*_*max*_(*n*) defined by local alignment of sequence *S* and matrix *Q*_*n*_(*i*) had the same distribution function. However, it is not possible to achieve complete identity of these distributions in the RPWM method. Therefore, to reduce errors in determining *n*_*max*_(*k*), we calculated *Z*(*n*) for each period length *n* using the Monte Carlo method; for this, steps 4–6 in the RPWM algorithm (section 2.1) were replaced with the new ones described below.

Step 4. For each TR of length *n*, we applied the Monte Carlo method to estimate statistical significance *Z(n)* of *V*(1): $Z(n)=(V(1)-\bar{V(1)})/(D{\left(V(1)\right)}^{0.5}$, where $\bar{V(1)}$ and *D*(*V*(1)) are the mean value and variance, respectively, of *V*(1) obtained by aligning random sequences *S* and matrix $Q_{n}^{\max}$.

Step 5. Steps 2–4 were performed for *n* from 2 to 50 to calculate all *Z*(*n*) values and determine *n* with the maximum *Z*(*n*) value, which was designated as *n*_*max*_(*k*), where coordinate *k* is the beginning of the window (sequence *S*) in the chromosome (step 1). Thus, for each window, we obtained *n*_*max*_(*k*), *Z*(*n*_*max*_(*k*)), matrix $Q_{n}^{\max}$, and local alignment of matrix $Q_{n}^{\max}$ and sequence *S*.

Step 6. Windows in sequence *S* were shifted by 10 bases to overlap with each other, and *Z*(*n*_*max*_(*k*)) values were filtered as follows: the dependence of *Z* on TR length (*n*) was excluded, resulting in *Z*(*n*_*max*_(*k*)) = *Z*_1_(*k*), and the local maxima in vector *Z*_1_(*k*) were chosen so that *Z*_1_(*k*) > *Z*_0_ and *Z*_1_(*k-i*) ≤ *Z*_1_(*k*)≥ *Z*_1_(*k+i*), where *i* ranges from 1 to 60. The selected local maxima represented the final result. Thus, for each local maximum, we obtained *n*_*max*_(*k*), *Z*(*n*_*max*_(*k*)), matrix $Q_{n}^{\max}$, and local alignment of matrix $Q_{n}^{\max}$ and sequence *S*.

#### 2.2.2. Consideration of the correlation of neighbouring bases

**Figure 1.**
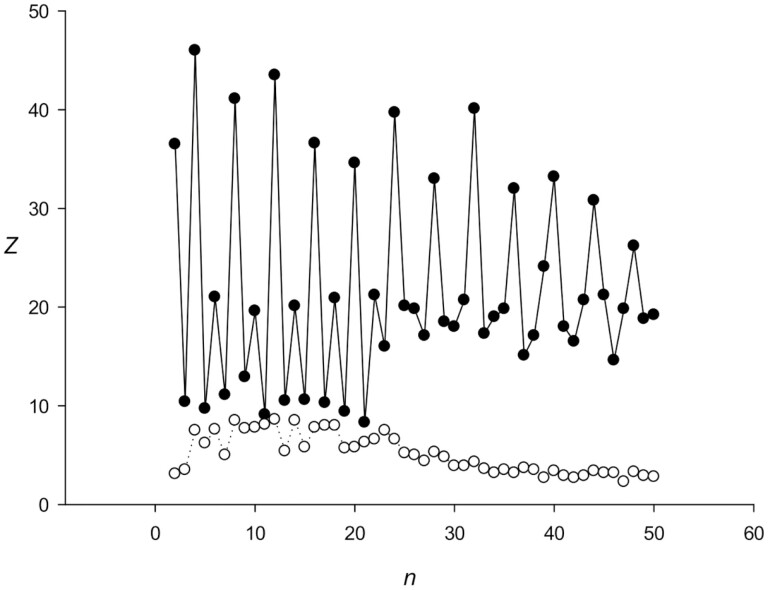
Calculation of statistical significance *Z*(*n*) depending on TRs length *n* for sequence *S*. White and black circles correspond to the results obtained by RPWM and mRPWM methods, respectively.

Matrix $Q_{n}^{\max}$ contains *n* rows and 4 columns (*n* × 4) and does not take into account the correlation of neighbouring nucleotides in sequence *S*, because the columns of the matrix are not interconnected; as a result, a significant part of TRs may remain undetected. To address this problem, we created an improved version of the RPWM algorithm, which should consider the correlation of adjacent nucleotides. For illustration, let us search for TRs with the length of 4 b (*n* = 4) in sequence *S* of length *L* = 1,200 b. To obtain *S*, we first randomly selected, with a probability of 0.25, one sequence *Seq*(*i*) (*i* = 1–4; *Seq*(1) = ATCG, *Seq*(2) = TAGC, *Seq*(3) = CCAA, and *Seq*(4) = GGTT) and filled in bases 1–4 in sequence *S*; the step was repeated until all the bases in sequence *S* (1–1,200) were filled. Then, we determined function *Z*(*n*) using the RPWM method.^33^ The results indicated that the 4 base TRs in *S* could be hardly seen (Fig. 1), because RPWM computed matrix *M*(*n*,4) for *Z*, which had *n* rows and 4 columns, i.e. for sequence *S*, at each position of repeat *n*, the frequency of each of the four bases (A, T, C, or G) should be the same and equal to 0.25.

**Figure 2.**
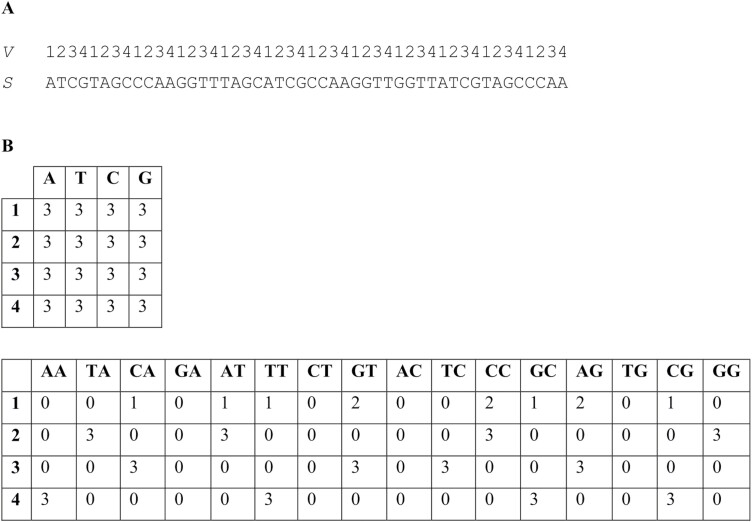
Illustration of the mRPWM method. Fig.2A This figure shows two sequences. The upper sequence contains the row numbers for the matrix *M*(*n*,4) for the case *n*=4 (sequence *V*). The lower sequence is the sequence *S* being studied. We start filling the matrix *M*(4,4) by moving from left to right along the sequence *V*. The first pair 1A will correspond to the first row and the first column of the matrix *M*(4,4) and then we add 1 to the cell (1,A). The second pair is 2T, it corresponds to cell (2,T), in this cell we add 1. So we move along the sequence *V* to its end *v*(*L*), where *L* is the length of the sequence *S*. As a result, we get a filled matrix *M*(4,4), which is shown in Fig. 2B. We can see that in this case the matrix *M*(4,4) is evenly filled and there will be no correlation between rows and columns. Fig. 2B below shows the filled matrix *M*(4,16). It was filled similarly to the matrix *M*(4,4), only their pairs are taken into account instead of individual bases. For example, for *j*=2, *s*(*j*-1)=A and *s*(*j*)=T, and *v*(2)=2 then we add one to cell *M*(2,AT). Then we take *j*=3, *s*(*j*-1)=T and *s*(*j*)=C, and *v*(2)=3 then in cell *M*(3,TC) we add one and so on until *j*=*L*. Fig.2B shows that the matrix *M*(4,16) is unevenly filled, indicating the presence of a correlation between the sequence *V* and *S*.

An example of such sequence *S* shown in Fig. 2A indicated that the first column contains a number of A, T, C, and G equal to 3. The same frequencies were observed for the second, third, and fourth positions of the periods; the resulting matrix is shown in Fig. 2B. These data mean that there are no differences in base frequencies at different positions of the repeat, which makes it difficult to detect TRs in sequence *S* according to base frequencies in matrix *M*. Therefore, low *Z* values were obtained using the RPWM method (Fig. 1); they were not zero only because dynamic programming arranged indels in a certain way.

In contrast, after the application of the modified (m)RPWM algorithm, 4 b-long TRs were clearly visible and *Z*(4) had the largest value among all repeat lengths (Fig. 1), because in mRPWM, instead of matrix *M*(*n*,4) (Fig. 2B, top), we used matrix *M*(*n,*16), which had 16 columns corresponding to 16 nucleotides (Fig. 2B, bottom). The elements of matrix *M*(*n*,16) were calculated as *m*(*v*(*j*),*i*) = *m*(*v*(*j*),*i*) + 1 (where *j* ranged from 2 to *L*, *i* = *s*(*j* − 1) + 4(*s*(*j*) − 1), and *v*(j) was an element of sequence *V*), indicating that matrix *M*(*n*,16) considered correlations of adjacent nucleotides. Matrix *M*(4,16) for sequence *S* (Fig. 2B, bottom) was extremely heterogeneous, and many of its elements were equal to zero. Thus, the newly developed mRPWM could identify TRs of 4 b in sequence *S*, i.e. appeared to be more efficient in search of TRs than the original RPWM.

In mRPWM, we considered the correlation of neighbouring bases by creating a set of matrices *Q*_*n*_ with dimensions *n* × 16, which had *n* rows and 16 columns instead of *n* rows and 4 columns, as described in section 2.1. Step 1; in addition, the size of the window was increased from 650 to 1,200 b and that of the step – from 300 to 600 b. The length (*n*) of TRs to be detected was also increased: from 2–50 to 2–200 b. In this analysis, we searched for TRs of all sizes, including those that were multiples of 3 b. As a result, the number of detected TRs was significantly increased. Thus, RPWM could identify approximately 76,000 TRs in the genome of rice (*Oryza sativa*), whereas mRPWM – 908,072 TRs in that of *C.annuum*. Given that the sizes of the two genomes are approximately 3.75 × 10^8^ and 3 × 10^9^ b, the average numbers of identified TRs per 10^6^ b were 192 and 300, respectively, confirming the superior performance of mRPWM.

### 2.3. Effect of nucleotide substitutions on TR identification

Artificial sequences of 1,200 b, which contained TRs of 10 b, were generated through tandem multiplication of random 10 b segments by 120 times; then, 0, 100, 500, 1,000, 1,500, 1,800, 2,200, and 2,400 random base substitutions and 20 indels were introduced at randomly selected positions. These artificial sequences were denoted as *St*(*x*) (where *x* was the average number of substitutions per base between any two repeats and was equal to 0, 0.17, 0.85, 1.7, 2.5, 3.0, 3.7 and 4.0)^39^ and used to search for TRs with RPWM and mRPWM and determine *Z*(10) as a function of *x*.

## 3. Results

### 3.1. Analysis of artificial sequences *St*(*x*)

**Figure 3.**
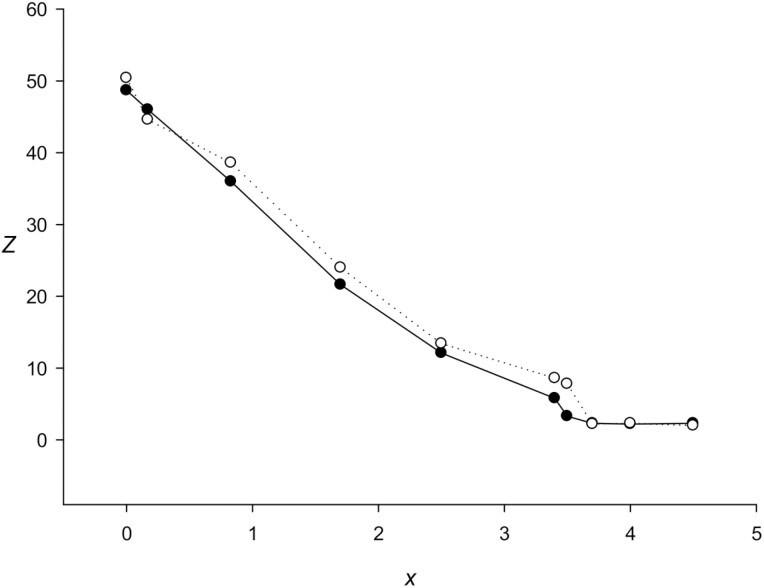
Dependence of statistical significance *Z*(*n*) on the number of base substitutions *x* for artificial sequences from set *St* (section 2.3). The continuous curve is the RPWM method, the dotted curve is the mRPWM method.

Figure 3 shows the plot of *Z*(10) versus *x*. If we take *Z*_0_ (section 2.1, step 6) equal to 6.0 (section 3.2), then RPWM can detect TRs with *x* up to 3.2, whereas mRPWM, which considers the correlation of neighbouring bases, can detect TRs with *x* up to 3.6, which significantly exceeds the limit for TRF and T-REKS methods: *x* < 1.2.^33^ Thus, the performance of mRPWM in identifying highly divergent TPs is superior to those of other currently used methods.

### 3.2. Detection of TRs in the *C. annuum* genome

Next, we applied the mRPWM method to search for TRs with lengths of 2–200 bp in the 12 chromosomes of *C. annuum* (sequences retrieved from Ensembl Plants databank, http://plants.ensembl.org/Capsicum_annuum/Info/Index). To select the significance level *Z*_0_, we searched for TRs in the first *C. annuum* chromosome and in a random sequence created by random mixing of chromosome nucleotides. The *Z* values for the sorted local maxima (section 2.1, steps 5 and 6) are presented in Table 1; among them, *Z*_0_ = 6.0 was chosen as the threshold level which provided a false discovery rate (FDR) of 2.71%. The number of TRs found in each *C. annuum* chromosome is shown in Table 2. Overall, 908,072 TRs were detected; their density was similar in all the chromosomes and constituted ~302 TRs per 10^6^ bp. The calculations were performed on two Ryzen 9 5950X processors, and the search for TRs in the *C. annuum* genome took about 2 weeks.

**Table 1.** Number of TRs found in the first *C. annuum* chromosome and random sequence

	*Z*_0_				
	5.0	5.5	6.0	6.5	7.0
First chromosome	161,367	129,788	97,727	72,578	54,306
Random sequence	33,033	10,494	2,651	681	198
FDR	20.47%	8.08%	2.71%	0.94%	0.36%

**Table 2.** Number of TRs in *C. annuum* chromosomes

Chromosome number	Chromosome size, bp	Number of found TRs
1	309,102,287	97,727
2	169,555,599	53,458
3	282,780,301	82,029
4	240,120,734	75,376
5	238,597,879	76,562
6	242,241,289	76,011
7	251,293,532	80,152
8	142,366,738	43,533
9	271,082,670	85,660
10	233,321,800	74,453
11	266,870,110	83,990
12	250,929,874	79,121
Total	2,898,262,813	908,072

**Figure 4.**
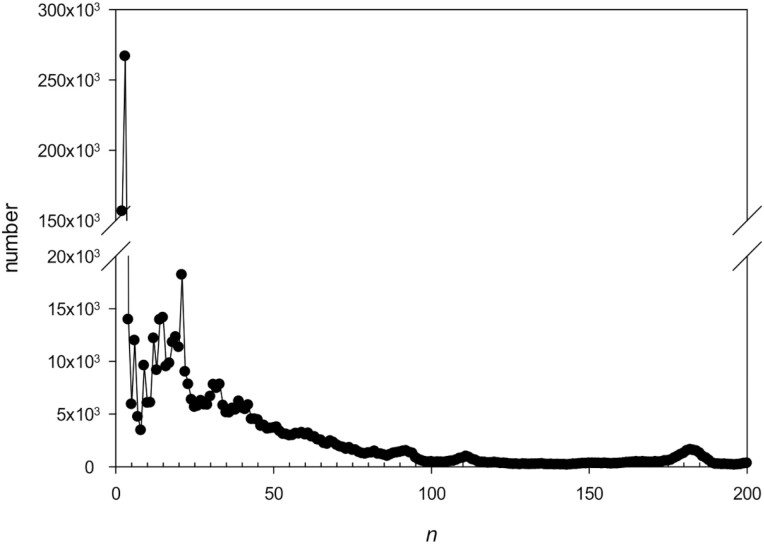
Frequency of TRs depending on the length *n.*

The statistics for the 10 most common repeat lengths indicated that TRs of 3 and 2 bp were the most frequent in the *C. annuum* genome (Table 3), which is consistent with the data reported for other plant genomes.^31,40^ Analysis of TR distribution depending on the length revealed that in addition to TRs with *n* = 2 and 3 bp, there were local peaks for those with *n* = 21, 31–33, 91–92, 111–112, and 178–187 bp (Fig. 4); the existence of lengthy TRs may be associated with chromatin compaction. It has been reported that in proteins, the most frequently observed repeat lengths are 2 and 7 amino acids,^41^ which correspond to 6 and 21 bp, respectively.

**Table 3.** Lengths of 10 most frequently identified TRs in the *C. annuum* genome

TR length, bp	Number of TRs
3	266,713
2	156,442
21	18,194
15	14,126
4	13,940
14	13,935
19	12,292
12	12,172
6	11,954
18	11,789

**Figure 5.**
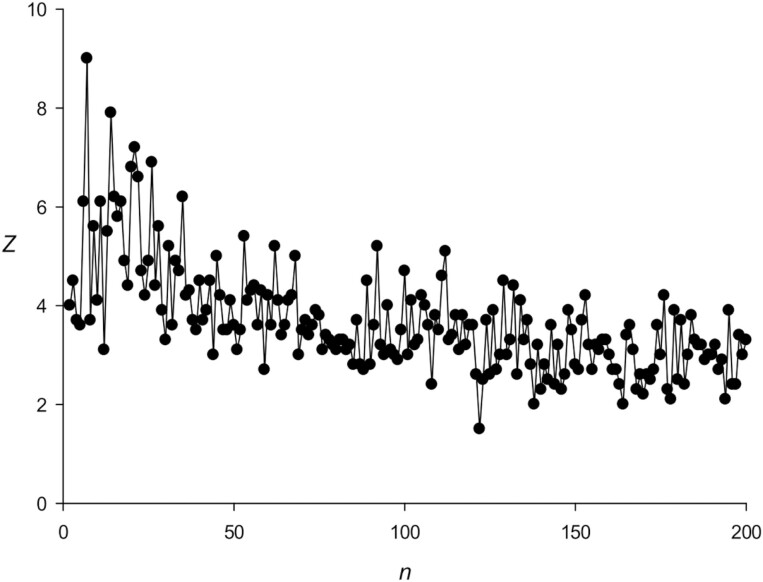
Statistical significance *Z*(*n*) for a fragment of *C. annuum* chromosome 1 (21,950,674–21,951,081 bp).

The performance of the newly developed mRPWM method was verified by searching for highly dissimilar TRs that could not be detected by the existing algorithms. TRs were analysed in a non-coding region of the first *C. annuum* chromosome (15,478,656–15,479,825 bp). The *Z*(*n*) plot indicated that a pronounced maximum (*Z* = 9.0) was observed for *n* = 7 bp and lower peaks were also detected for *n* = 14, 21, 28, and 35 bp (Fig. 5). Table 4 shows the alignment between a TR and an artificial periodic sequence containing 34 indels with a maximum length of 3 bp; the corresponding $Q_{7}^{\max}$ matrix is shown in Table 5.

**Table 4.** Alignment of TRs with $Q_{7}^{\max}$ shown in Fig. 5

AAGCACCACGTGTCAAATGACGTGGCATGCTAAGTCAAGAAA*TGAATCCAATAGGACCATGCCACATGTCAAAAT*GATGCAGCAGGCC 67123456712345671234567123*456712345671234567123456712345671234567123456712345671234567123
CATGAAATCAAAGTTCATAAAAGGTGTCATGTCACTCAAGTATGATTGGTCAAAGAAAGTCCATTTTCATCAT*GACTCTTCCCTTTCCA 45671234567123456712345671234567123456712345671234567***1234567123456712345671234567123456
CAACTATAAATAGGGGGCCCATAATTCAGAAAAGAAGCTCAGAACTCTAAAAGCAGCAAGAGAAAGCTCGTGGATCAAACGCCACAATTT 712345671234567123456712345671234567123456712345671234567123456712345671234567123456712345
TCCTAAAAAGCTCAAGCATTCAAGTCAAGTTCATCAAGATCCAAAATCAAGACCACAATATTCAAAAACAAGCTCAAAAGCCCTTGAATT 671234567123456712345671234567123456712345671234567123456712345671234567123456712345671234
CAAGC***ACAAGTCAAGAT*CAAGTCCCCCAAATCAACAAATCAAGTTCAA*ATTCAAGAT*CAAGCTTCAAACCCTTGAATTTATATT 567123456712345671234567123456712345671234567123456712345671234567123456712345671234567123
TGAAAAGGCGAATTAGAAGATTCATAGAGATTGTAACACTCACATATTGAAATCAATAAATTGATTGTTTAATATTTTCTTGGCTCAATT 456712345671234567123456***712345*67123456*712345671234567123456712345671234**567123456712
ATTTATTTTTTCGATCCCAAAAATTTTATTGTCCAACAAATTCTGGCACGCCCAGTAGGACAATCTCTATCTGTCATCTCAACTGCTCCA **3456712345671234567123456712345671234567123456712345671234567123456712345671234567123456
AC*TGCAAAGTTCAACAACACTGAAATGACTTCCAAGAAGGTCAACTCTCAATCAACTACATCTAAGGCTGCTGATTCAAAGTTCTCTGG 7123456712345671234567123456712345671**234567123456712345671234567123456712345671234567123
TGAAGTAGAAAGCATCCTTGGTGTTATTTTCGAAAGCTTAGGAACTGTCACAAAGAGCAAGGAAGG**CTTGCTAGGACAACAAACACAT 456712345671234567123456712345671234567123456712345671234567123456712345671234567123456712
CCAGTGTCGTCCGAACCAACTCCAATTTTTGAATCTTCAACCCCAAAAGGAAAGAAATTCAATGCAAGTTCTTCGGAAGGAGGAAGCAGT 34567123456712345671234567123456712345671234567*123456712345671234567123456712345671234567
GTGGCGGAAT*CGCTTAAGAAGACTCTAGATTTA**CTTGAGAATTCCAGTTCCAAACACTCTGGCACAAAGAGCAATGATCGTTCGAGC 123456712345671234567123456712345671234567123456712345671234567123456712345671234567123456
AACTCATCATCTCCGACTATACCGCATAAGTTGAGCGCTTCAAAGATCAACTTGTGCGATAATCCATGCTACTTTCCGATATCTTCAGTG 712345671234567123456712345671234567123456712345671234567123456712345671234567123456712345
ATTATGCAAGTGATGGTGACTGATGCCTCGTCTATGAAGGAGCA*GCTTGAGAATTTAGCAAAGGCAATTAAGAGCCTGACCAAATATGT 671234567123*45671234567123456712345671234567123456712345671234567123456712345671234567123
TCAGAAT*CAAGATGC 4567123456712345

^*^indicates indels

**Table 5.** $Q_{7}^{\max}$ matrix for the alignment built in Table 4

	AA	TA	CA	GA	AT	TT	CT	GT	AC	TC	CC	GC	AG	TG	CG	GG
**1**	−0.7	−0.1	0.2	5.0	−3.0	−0.4	−0.6	2.4	−1.9	1.6	−1.6	−0.5	0.8	−2.2	−1.2	−1.3
**2**	0.4	−1.1	−1.2	0.5	−3.8	−1.0	−0.3	−0.1	−1.7	0.6	−2.0	−1.6	0.1	1.6	2.9	1.6
**3**	−3.6	4.9	−2.0	−0.1	−3.6	0.5	1.0	0.6	−2.5	1.8	0.0	−1.7	−1.7	−0.5	1.0	0.7
**4**	−3.2	−1.1	−1.9	−1.2	−4.0	8.9	1.4	−0.4	−1.9	0.6	1.1	−1.6	−2.2	1.7	−0.8	−0.0
**5**	−3.9	−2.4	−1.1	−0.3	−4.3	−0.9	12.3	3.5	−0.8	−0.6	1.5	−2.0	−2.8	1.8	2.2	−1.7
**6**	−4.2	−1.7	−2.3	−0.1	−2.8	−1.9	−1.5	−1.1	11.1	−0.7	0.0	0.9	1.0	−1.9	−0.9	−0.1
**7**	7.5	−0.6	−0.9	−0.0	−3.3	−1.3	−1.5	−1.1	−3.3	−0.4	−1.3	−1.3	−2.2	1.6	−0.5	−0.5

Supplementary material files Period_5.tif and Period_21.tif show the results for two other regions of the first *C. annuum* chromosome: 21,950,674–21,951,081 bp and 5,039,380–5,040,582 bp, in which periodicity of 5 and 21 bp, respectively, was detected; the corresponding matrices $Q_{5}^{\max}$ and $Q_{21}^{\max}$ can be found by coordinates in the database at http://victoria.biengi.ac.ru/capsicum_tr/. We also constructed multiple alignments for the three analysed regions of the first *C. annuum* chromosome (Supplementary material files Multi7.txt, Multi5.txt, and Multi21.txt).

Cumulatively, these results indicate that the mRPWM method can identify such TRs that cannot be detected by the other existing algorithms.

### 3.3. Presence of TRs in the annotated *C. annuum* sequences

Next, we analysed the distribution of the detected TRs in the *C. annuum* genes (http://ftp.ensemblgenomes.org/pub/plants/release-53/gff3/capsicum_annuum/), potential promoter sequences (PPSs; http://victoria.biengi.ac.ru/cgi-bin/dbPPS/index.cgi), and SINE repeats (http://victoria.biengi.ac.ru/sine_pepper/index/) by calculating the number of TRs that overlapped with any annotated sequence by at least 50%. The results indicated that the number of TRs intersecting with the genes was 42,210, which exceeded that of the *C. annuum* genes (31,600), i.e. one gene contained several TRs. The number of genes overlapping with TRs was 20,739, indicating that approximately 2/3 of the annotated *C. annuum* genes contained repeated sequences. Among these genes, 80% (16,656) had TRs with repeat lengths that were multiples of 3 bp (including 14,275 with the length of 3 bp), which is consistent with a previous report that over half of all genes have 3 bp TRs.^42,43^

Among the 825,136 PPSs^44^ and 50,077 SINEs (unpublished data) found in the *C. annuum* genome,^44^ 277,929 and 7,444, respectively, contained TRs, among which those of 2, 3, and 21 bp long were the most prevalent, corresponding to the overall statistics of TR length distribution in the genome (Table 3).

### 3.4. Cluster analysis of TRs with lengths 2 and 21 bp

Among the most common TRs in the *C. annuum* genome (Table 3), those with *n* = 3 bp have been studied in detail^42,43^; therefore, we focused on TRs with *n* = 2 and 21 bp and analysed their heterogeneity, i.e. similarity of TRs from different regions. For this, we performed cluster analysis of *n* × 16 matrices $Q_{n}^{\max}$ for *n* = 2 and 21 bp (section 2.1, step 6), in which matrix $Q_{n}^{\max}$ was considered as a point in the Euclidean space *n* × 16, and determined the difference between two matrices (designated as $m_{}^{1}(i,j)$ and $m_{}^{2}(i,j)$) according to the Euclidean distance. As a TR-containing region can begin at any repeat position, all variants should be taken into account for cyclic shifts between matrices. In the example presented in Fig. 2A, sequence *V* started from the first position, which was arbitrary, because it could start from any position in the repeat (for example, 3412341234…). Therefore, to calculate *Dist*^*n*^, which is the smallest distance between two matrices, we cyclically shifted the beginning of one of them by 0, 1, 2, …, *n* − 1 positions so that all possible options for matching matrices were considered. In total, there were *n* distances between two matrices:


$$Dist^{n}\;=\;\underset{t}{\min}\;\left\{\sqrt{\sum_{i}^{n}\sum_{j}^{16}}\;(m^{1}(i,\;j)\;-\;m^{2}(k(t),\;j))\right\}$$ (1)

where *k*(*t*) = *i + t* − *n*{*int*((*i* + *t* − 0.5)/*n*)}.

The minimum was found among all values of *t* from 0 to *n* − 1. By determining *Dist*^*n*^ between all pairs of matrices, we built a matrix of paired distances for all matrices $Q_{n}^{\max}$ obtained for a repeat of length *n* and then applied the hierarchical clustering algorithm. However, 156,442 and 18,194 TRs of 2 and 21 bp, respectively, resulted in very large distance matrices; therefore, to reduce matrix dimensions, we grouped 2,000 randomly selected matrices $Q_{n}^{\max}$ into sets *W*_2_ and *W*_21_ and performed clustering using the statistical package R and the Complete Linkage algorithm.

**Figure 6.**
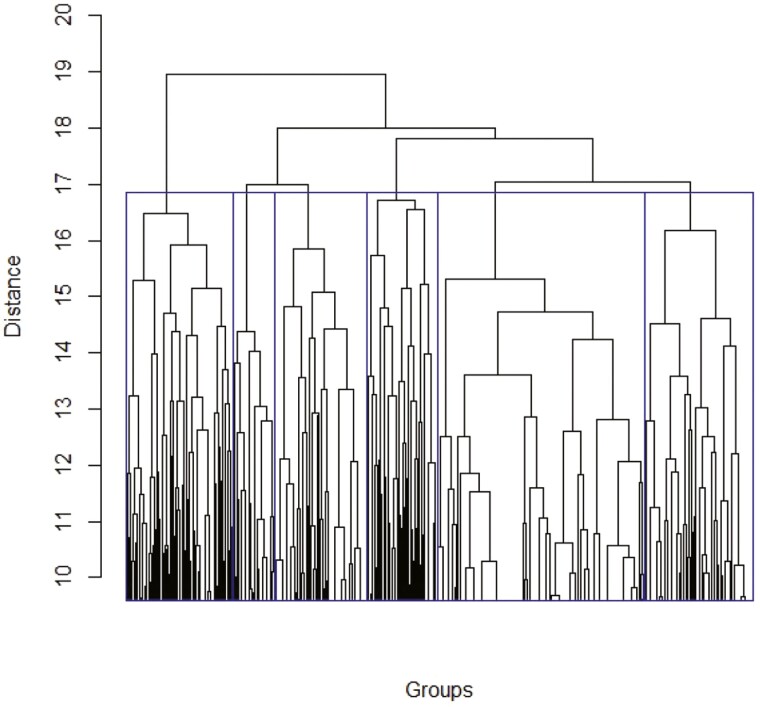
Cluster dendrogram of 2,000 random TRs with the length of 2 bases in the *C. annuum* genome. Blue rectangles indicate TR clusters for *Dist*^2^_0_=17. As the number of matrices for clustering was large (2,000), the plot is truncated for clarity of presentation.

**Figure 7.**
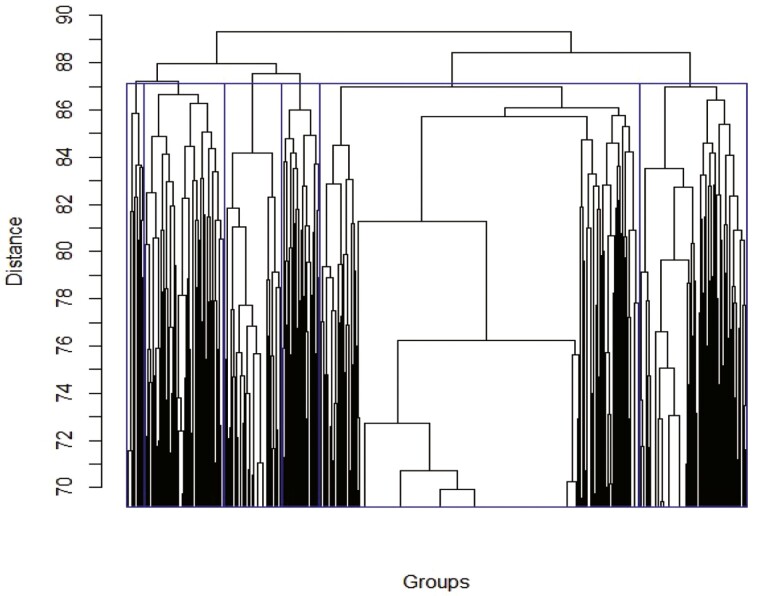
Cluster dendrogram of 2,000 random TRs with the length of 21 bases in the *C. annuum* genome. Blue rectangles indicate TR clusters for *Dist*^21^_0_= 87. As the number of matrices for clustering was large (2,000), the plot is truncated for clarity of presentation.

The results are shown in Figs 6 and 7. Distance values $Dist_{0}^{2}$ = 17 (*n* = 2) and $Dist_{0}^{21}$ = 87 (*n* = 21) were taken as group-forming levels. To select $Dist_{0}^{n}$, we randomly shuffled the cells in each matrix of sets *W*_2_ and *W*_21_ to obtain sets of random matrices $WR_{2}^{i}$ and $WR_{21}^{\;j}$ (*i*, *j* = 1…100) and subjected them to cluster analysis, which produced 100 random matrix clusterings per set. Then, each clustering was analysed for the number of classes and matrices in each class at levels $Dist_{0}^{2}$ = 17 and $Dist_{0}^{21}$ = 87. For random set $WR_{2}^{i}$ (*i* = 1…100), the average number of matrices in the class (${\bar{N}}_{2}$) was 43.8 and variance *D*(*N*_2_) was 3.88. The statistical significance of creating classes was determined as $Z_{n}={\left(N_{n}-{\bar{N}}_{n}\right)/\left(D(N_{n})\right)\;}^{0.5}$. At $Dist_{0}^{2}$ = 17, there were six classes (*N*_2_ = 6) containing 339, 135, 293, 224, 663, and 346 matrices, respectively, and *Z*_2_ for each class was > 20.0. For random set $WR_{21}^{\;j}$ (*i* = 1…100), ${\bar{N}}_{21}$ = 6.1 and *D*(*N*_21_) = 0.009. At $Dist_{0}^{21}$ = 87, *N*_21_ = 6, the number of matrices in each class was 57, 258, 185, 124, 1028, and 348, respectively, and *Z*_21_ for each class was also > 20.0. Class matrices for TRs with repeat lengths of 2 and 21 bp are given in Supplementary materials (groups2_17.txt and groups21_87.txt, respectively).

### 3.5. Database of *C. annuum* TRs

The TRs detected in the *C. annuum* genome are placed into a database (http://victoria.biengi.ac.ru/capsicum_tr/), which provides the following information for each TR-containing region: chromosome number, repeat length *n*_*max*_, Z(*n*_*max*_), coordinates in the chromosome, sequence, matrix $Q_{n}^{\max}$ and local alignment of the matrix. The available sequence annotation is also shown if it overlaps by at least 50% with a TR-containing region. Site http://victoria.biengi.ac.ru/splinter/login.php provides the application to search for TRs in DNA sequences using the improved algorithm mRPWM.

## 4. Discussion

In this study, we modified the RPWM method developed earlier^33^ so that the new version, mRPWM, could detect TR regions with much longer repeats (2–200 bp) than the original RPWM (2–50 bp). mRPWM is a novel method which detects TRs using only the DNA sequence and does not require a library of previously defined TRs as is the case with HipSTR.^45^

To compare the performance of mRPWM and the original RPWM, we applied them to search for TRs in the *C. annuum* genome. In the first *C. annuum* chromosome, RPWM could detect 69,546 regions with different period lengths, whereas mRPWM detected 97,727 of such regions (Table 2), which is about 40% more. For the period length from 2 to 50 bp, mRPWM detected 82,427 TRs, indicating that 15,300 regions identified by mRPWM contained repeats with the length between 51 and 200 bp.

To match TRs found by RPWM and mRPWM, we analysed their intersection, i.e. the ratio of the total length of the two TR-containing regions to the length of the smallest one (Table 6). The results revealed that 46,093 regions (67% of those detected by both methods) overlapped considerably (intersection *C* > 0.5), whereas 7,803 (~11%) overlapped weakly, and 15,560 (~22%) were unique, indicating consistency between the results obtained by the two RPWM methods. However, the coincidence of period lengths in the overlapping areas constituted only ~11% (8,047 regions), which could be attributed to the following reasons.

**Table 6.** Number of intersecting regions containing TRs found by RPWM and mRPWM methods. The first row shows the degree of intersection (*C*), and the second row the number of regions with a given degree of intersection (*N*)

*С*	0–0.1	0.1–0.2	0.2–0.3	0.3–0.4	0.4–0.5	0.5–0.6	0.6–0.7	0.7–0.8	0.8–0.9	0.9–1.0
*N*	1,538	1,494	1,469	1,681	1,711	1,864	2,043	2,315	2,833	37,038

First, to estimate the statistical significance of the identified TRs, RPWM uses *Vec*(*k*), which contains *F*_*max*_ and which is calculated by dynamic programming (section 2.1, step 3) and must be greater than threshold value *V*_0_.^33^ The local maximum is determined using dynamic programming and the PWM, and the distribution function for *F*_*max*_ strongly depends on TR length *n* and PWM *mQ*,^33^ which means that at the same *F*_*max*_ value, probability *P*(*F* > *F*_*max*_) may differ for different *n* and *mQ* despite special transformations of matrix *mQ*.^33,37^ To speed up the calculations, among *n* = 2–50 bp we have chosen *n* with the largest *F*_*max*_.^33^ However, it is not quite correct to do so when the distribution function for *F*_*max*_ depends on *n*; in this case, *n* with the lowest probability *P*(*F* > *F*_*max*_) should be chosen. To estimate *P*(*F* > *F*_*max*_) in this study, we used the Monte Carlo method for calculating statistical significance *Z*(*n*) (section 2.2.1, step 4), which allows a more accurate determination of the period length for TRs and which accounts for the differences in period lengths observed in the overlapping regions.

Second, in the intersecting regions, there are periods *t* for which *t*/*n*_0_ is an integer equal to or greater than 2 (here, *n*_0_ is the length of TRs). *Vec*(*k*) for *n* = *t* (section 2.1, step 5) may have the maximum value among all periods, whereas *Z*(*n*) (section 2.2.1, step 4) may be maximal for *n* = *n*_0_ because of weak nucleotide correlation at *n* ≥ *n*_0_. Therefore, the lengths of TRs identified by the RPWM and mRPWM methods would be different. As an example, we used an artificial sequence {ATCGATTCGG}_120_ (1,200 nt long), in which 1,200 random substitutions were introduced at random positions (Supplementary material, file seq_period_10.txt); for this sequence, *n*_*max*_(*k*) = 20 bases and *Vec*(*k*) = 2,480, whereas *n*_0_ = 10 bases and *Z*(*n*_0_) = 11.8 (full ranges of *Z*(*n*) and *Vec*(*k*) values depending on period length *n* are shown in Supplementary material files 10.new and 10.old). It appeared that RPWM and mRPWM showed different period lengths if the number of accumulated base substitutions (*x*) was large and that mRPWM could determine *n*_0_ more accurately.

Third, it is possible that in some TRs a short period *t* is nested in a longer period *n*_0_ without dividing the latter. In this case, RPWM can detect TRs of length *n*_*max*_(*k*) = *t*, whereas mRPWM can find those with length *n*_0_ because of base correlation. Let us illustrate it on an example of sequence {ATCGATCGATCGATCGCGG}_57_ which simultaneously contains both short and long periods of lengths 4 and 21 bases, respectively, and in which we made 1,200 random substitutions in random places (Supplementary material file seq_period_21.txt.). As a result, *n*_*max*_(*k*) = 4 and *Vec*(*k*) = 2,634, whereas *n*_0_ = 21 and *Z*(*n*_0_) = 9.2 (full ranges of *Z*(*n*) and *Vec*(*k*) values are shown in Supplementary material files 21.new and 21.old). Thus, mRPWM, by taking into account the correlation of neighbouring nucleotides, is able to detect much longer periods than the original RPWM.

Pepper was chosen for analysis as a popular vegetable crop widely cultured around the world, whose genetic structure and properties are of interest to agriculture, the food industry, and medicine. It is known that 81% of the *C. annuum* genome are repetitive sequences, of which 76.4% are transposons,^46^ and more than 70% of the latter are long terminal repeat elements. The pepper genome has been previously analysed for the presence of dispersed repeats and transposable elements^34^; however, there are no data on TRs. Using mRPWM, we identified 908,072 TRs with an average length of 917 bp, indicating that TRs occupy about 29% of the *C. annuum* genome. However, this number is a minimum estimate, because we targeted regions containing > 4 repeats with a length of 2–200 bp. Most TRs had a repeat length of 2 or 3 bp; the latter may be partly related to coding sequences,^47^ because 2/3 of the genes contain TRs, of which 80% have a repeat length multiple of 3 bp. In contrast, 2 bp TRs are mostly found in non-coding regions and only 4.6% of them intersect with the genes. TRs with a repeat length of 21 bp are also relatively numerous in the *C. annuum* genome; it is suggested that weakly similar TRs with the length 10.5 bp (10.5 × 2 = 21 bp) may play a role in DNA packaging within chromatin^19^ or be involved in the formation of alpha helices in proteins.^48^

Cluster analysis of TRs with *n* = 2 or 21 bp resulted in the identification of several groups and the creation of an averaged matrix for each. Such matrices could be used to find weakly similar TRs of a certain type using hidden Markov models or dynamic programming, which may be important for evolution studies.

A large number of algorithms have been developed for TR search in DNA and proteins.^22–25,29,49^ Some of them perform best in finding short perfect repeats, whereas the others are focused on weakly similar TRs with a relatively long period.^41^ Comparison with the other methods such as TRF,^22^ T-REKS,^24^ and XSTREAM^25^ could be useful for assessing the performance of mRPWM. However, different methods use various input parameters, which could affect the number of detected TRs^50^; therefore, it is necessary to apply a common criterion of TR recognition for objective comparison. As in mRPWM we used the FDR (obtained by analyzing real and random DNA sequences) and searched for TRs in a 1,200 b window that moved along the chromosome with a step of 600 b, the same parameters were applied to compare the performance of the other algorithms with that of mRPWM in identifying TRs in the first *C. annuum* chromosome. Fragments from 600*i* + 1 to 1200 + 600*i* (*i* = 0.1…) were cut out, windows containing character N were removed, and the resultant windows were designated as set *Win*; then, the three methods mentioned above were used to detect TRs in sequences from set *Win* and to determine local maxima (section 2.1, step 6). To estimate the FDR, TRs were also searched in random sequences of set *WinR* obtained by random mixing of nucleotides in all windows from set *Win*. Various parameters of TRF and T-REKS were considered: for the former, we changed the alignment weight and for the latter, we took a different psim; the aim was to achieve FDR < 6.0%, which is not much higher than that for mRPWM (FDR = 2.71%, Table 1). XSTREAM was started with default settings. The detected TR regions were filtered to contain at least four repeats with a length of 2–200 bp in order to correspond with mRPWM search parameters. The results indicated that T-REKS, regardless of the psim parameter, could not find TRs with the FDR < 6.0%, whereas XSTREAM detected only 16,520 TR regions with more than 4 repeats at the FDR ~ 6.0%, and TRF found a total of 28,245 TRs (FDR = 2.82). mRPWM for number of repeats ≥ 4 identified 97,727 TRs at the FDR = 2.71% (Table 1). The reason for such a noticeable difference is that mRPWM does not look for similarity between individual repeats but uses an image of multiple alignments of all TRs.

We also compared the performance of mRPWM with those of GMATA and TANTAN. GMATA is a fast method to search for microsatellites in large genomes,^51^ and TANTAN is a program to identify low-complexity regions and STRs, which are then excluded from consideration in further sequencing studies to avoid their contribution to noise. First, we determined *x* values for which TRs could be detected in artificial sequences (section 2.3) and found that GMATA and TANTAN could detect TRs and low-complexity regions only for *x* ≤ 0.17 and *x* ≤ 0.85, respectively, but could not identify STRs with higher *x*. Then, we applied the two programs to search for TRs detected by mRPWM. For this purpose, we created a set of 10^4^ randomly selected sequences (*Set*_*m*_) with length *l*(*i*) (*i* = 1, 2,…, 10^4^), which contained the TRs identified by mRPWM in the *C. annuum* genome. GMATA found only five *Set*_*m*_ sequences with a length greater than 50% of the corresponding *l*(*i*); in these cases, TR lengths were the same or multiples of those detected by mRPWM. In addition, GMATA found many (5,122) short sequences with an average length of 11.5 nt (< 2% of that of *Set*_*m*_ sequences) and *n* = 2–5 nt. These results indicate that GMATA is unable to identify the majority of TRs found by mRPWM and finds only their small fragments, which is likely due to the fact that the program can recognize only TRs with very few base substitutions (*x* ≤ 0.17). Often, GMATA is followed by HipSTR which genotypes STR alleles through comparison of the genome sequences of multiple polymorphic variants^52^; however, HipSTR analyses only TRs found by GMATA or other *de novo* methods and cannot identify the repeats missed by these methods, indicating that most TRs detected by mRPWM also cannot be found by combining GMATA with HipSTR.

A similar situation was observed for TANTAN, which in *Set*_*m*_ identified only 8,191 short sequences with an average length of 34.6 nt and only 411 regions with a total length greater than 50% of the corresponding *l*(*i*). Thus, TANTAN, similar to GMATA, could not find most TRs detected by mRPWM and recognizes only small fragments.

We have already applied mRPWM to multiple alignments of promoter sequences^53^ and TR search in the *O. sativa* genome.^33^ Here, the method was improved to take into account the correlation of neighbouring nucleotides, which resulted in the increase of TR density per 10^6^ bp from 192 (*O. sativa* genome) to 302 (*C. annuum* genome), i.e. by > 1.5 times. Thus, the mRPWM method developed in this study shows superior efficiency in identifying highly divergent TRs with a wide range of repeat lengths in DNA sequences, which is important for functional and evolutionary analyses of the genomes.

## Supplementary Material

dsad007_suppl_Supplementary_Data_S1

dsad007_suppl_Supplementary_Data_S2

dsad007_suppl_Supplementary_Data_S3

dsad007_suppl_Supplementary_Data_S4

dsad007_suppl_Supplementary_Data_S5

dsad007_suppl_Supplementary_Data_S6

dsad007_suppl_Supplementary_Data_S7

dsad007_suppl_Supplementary_Data_S8

dsad007_suppl_Supplementary_Data_S9

dsad007_suppl_Supplementary_Data_S10

dsad007_suppl_Supplementary_Data_S11
