# Supplementary figures and images for: Detection of tandem repeats in the Capsicum annuum genome
Source: DNA Res. 2023 Apr 25;30(3):dsad007. doi: 10.1093/dnares/dsad007 (PMC10211496; doi:10.1093/dnares/dsad007)

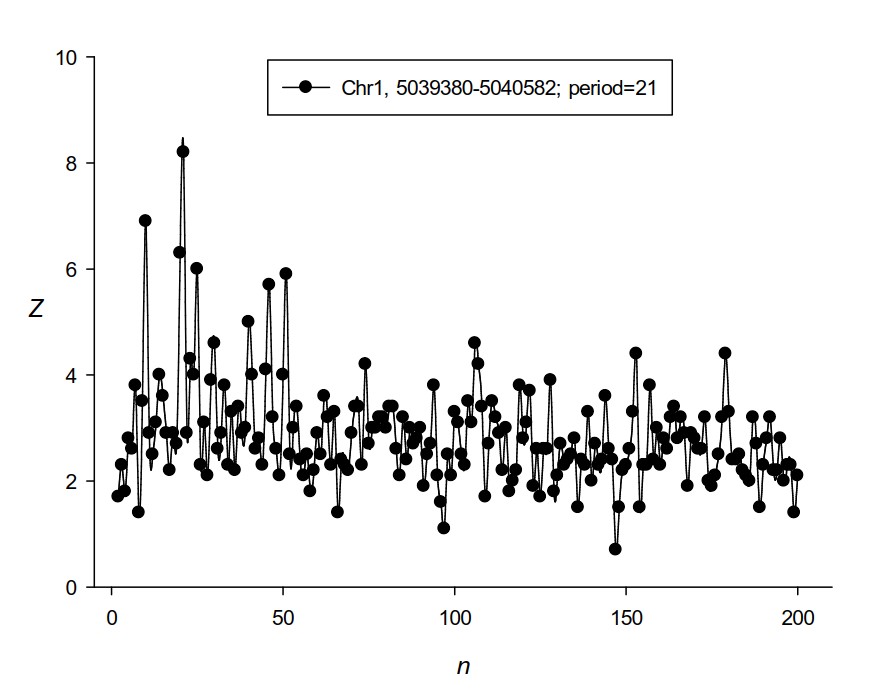

Supplement: dsad007_suppl_Supplementary_Data_S10 [file dsad007_suppl_supplementary_data_s10.jpeg]

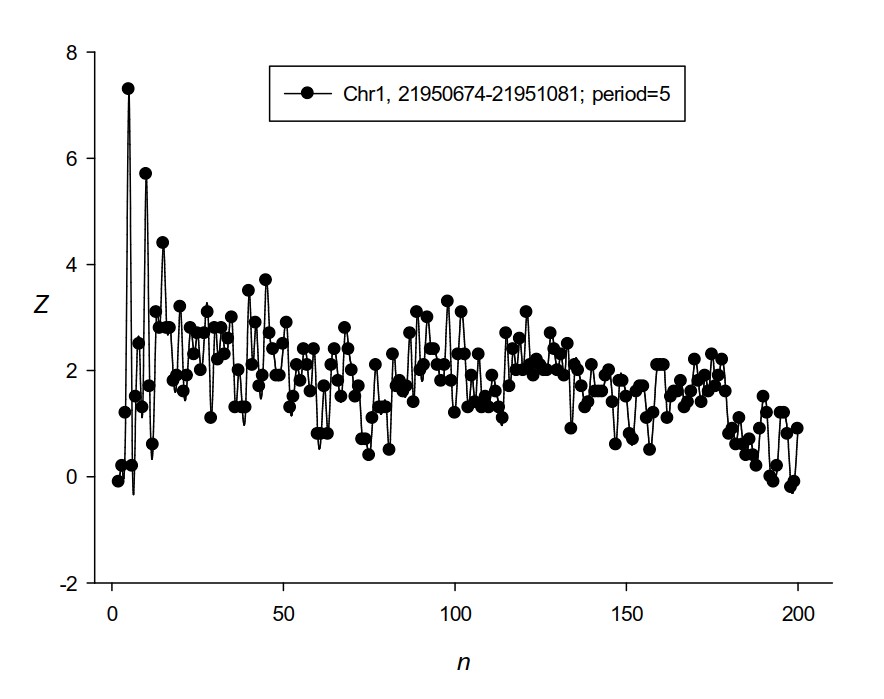

Supplement: dsad007_suppl_Supplementary_Data_S11 [file dsad007_suppl_supplementary_data_s11.jpeg]
